# Circadian clock genes and insomnia: molecular mechanisms and therapeutic implications

**DOI:** 10.1080/07853890.2025.2576643

**Published:** 2025-10-22

**Authors:** Qing Ren, Mingyi Gu, Xu Fan

**Affiliations:** ^a^ Liaoning University of Traditional Chinese Medicine Affiliated Hospital, Shenyang, China; ^b^ Liaoning University of Traditional Chinese Medicine, Shenyang, China

**Keywords:** Circadian clock genes, insomnia, molecular mechanisms, therapeutic targets, pharmacology

## Abstract

**Background:**

Insomnia, affecting 30–40% of the global population, is a debilitating sleep disorder linked to significant health risks, including cardiovascular disease, metabolic syndrome, and neurodegeneration. Emerging evidence implicates dysregulation of circadian clock genes as a core molecular mechanism underlying its pathophysiology.

**Methods and results:**

This review synthesizes current knowledge on how core clock genes regulate the sleep–wake cycle *via* transcription–translation feedback loops, incorporating recent insights into regulatory layers such as SUMOylation. We discuss how genetic polymorphisms and epigenetic modifications disrupt circadian rhythmicity, predisposing individuals to insomnia. The molecular pathways linking clock dysfunction to insomnia encompass dysregulation of neurotransmitter systems (melatonin, serotonin, GABA, dopamine), metabolic imbalance, neuroinflammation, mitochondrial oxidative stress, and altered synaptic plasticity. Chronic circadian misalignment, often driven by aberrant light exposure, exacerbates these disruptions.

**Therapeutic implications:**

Targeting circadian pathways presents novel therapeutic avenues. Melatonin receptor agonists facilitate sleep initiation and phase alignment; synthetic REV–ERBα/β ligands enhance circadian amplitude; dopaminergic modulators address hyperarousal; and GABAergic drugs restore inhibitory balance. Notably, Traditional Chinese Medicine formulations exhibit multi-pathway regulatory effects on clock gene expression. However, treatment efficacy varies across insomnia subtypes, and challenges regarding pharmacokinetics and long-term safety remain.

**Conclusion:**

Dysfunctional circadian clock genes are pivotal in insomnia pathogenesis *via* interconnected molecular pathways. Future research should focus on biomarker-driven, personalized chronotherapies targeting these genes and their downstream effects to improve clinical outcomes.

## 1. Introduction

Insomnia is a common sleep disorder, which affects about 30–40% of the general population in the world. Its main manifestations are difficulty in falling asleep at night, difficulty in maintaining sleep or waking up early [1]. Long-term insomnia not only impairs sleep quality, but also has a significant impact on daily functioning and overall health, increasing the risk of cardiovascular disease, metabolic disorders, and mental health problems such as depression and anxiety [2]. In addition, insomnia may also induce digestive and respiratory diseases and metabolic syndrome, while increasing the incidence of neurodegenerative diseases such as mild cognitive impairment and dementia [3]. These factors have made insomnia increasingly attracting attention from the medical community.

Insomnia can be divided into two types: primary and secondary. Primary insomnia refers to simple insomnia that still exists after excluding physical diseases, mental disorders and drug effects, there is no clear cause at present [4]. Secondary insomnia is caused by other diseases, such as depression, anxiety, diabetes and cardiovascular disease [5,6]. Despite the high prevalence of insomnia and its significant health consequences, its underlying molecular mechanisms are still not fully understood, emphasizing the need for further research to identify new treatment targets.

In recent years, advances in chronobiology have revealed the key role of biological clock genes in regulating the sleep–wake cycle and maintaining overall physiological homeostasis [7]. The biological clock gene is the molecular basis of the endogenous circadian rhythm. External light is transmitted to the suprachiasmatic nucleus (SCN) through the retina–hypothalamic tract, forming a series of transcription and translation feedback loops. These circuits synchronously regulate the expression of the peripheral biological clock through the interaction and periodic expression of biological clock genes, thereby keeping the body synchronized with the outside world by alternating day and night [8]. The core transcription and translation feedback loop is mainly composed of transcription factors Brain and Muscle ARNT-Like 1/Circadian Locomotor Output Cycles Kaput (BMAL1/CLOCK), Period Circadian Regulators (PERs) and Cryptochromes (CRYs). BMAL1 and CLOCK act as activators of the biological clock, forming heterodimers and activating the transcription of PERs and CRYs genes [9]. PERs and CRYs are photoreceptor genes that act as inhibitors of the biological clock and form a positive and negative feedback loop with BMAL1 and CLOCK [10]. Studies have found that mutations in the BMAL1 gene can lead to increased conversion from non-rapid eye movement sleep (NREM) to rapid eye movement sleep (REM), shortened deep sleep time, reduced sleep, and increased night activity, which in turn leads to changes in diet patterns, glucose intolerance and liver steatosis [11,12]. Mutations in the CLOCK gene can lead to reduced sleep time, continued nerve excitement, advanced phase, and circadian rhythm disorders [13]. Mutations in the PERs and CRYs genes can also affect sleep. For example, mutations in the PERs gene impair the body’s ability to maintain the stability of the circadian cycle, shortening the circadian cycle [14]. Mutations in the CRYs gene can reduce the number of sleep awakenings in mice, increase NREM sleep time and electroencephalogram (EEG) slow-wave activity [15]. In addition, genetic variants in clock genes such as PER3 and CRY1 have been linked to changes in sleep patterns and increased susceptibility to sleep disorders [16]. These findings highlight the potential importance of biological clock genes in the pathophysiology of insomnia and suggest that targeting these genes may provide new treatment opportunities.

The primary objective of this review is to delve into the molecular mechanisms linking circadian clock genes to insomnia. We place a strong emphasis on understanding how these genes regulate the neurotransmitter systems and cellular signaling pathways that are vital for sleep regulation. Furthermore, we explore the clinical significance of these findings, particularly the potential of circadian clock genes as targets for insomnia treatment. By integrating insights from molecular biology, neuropharmacology, and clinical research, this review aims to enhance our comprehension of insomnia’s pathophysiology and to identify promising avenues for future research and therapeutic development. It should be noted that the molecular mechanisms discussed herein, while particularly relevant to primary insomnia, also extend to secondary insomnia. This is because circadian misalignment, neurotransmitter dysregulation, and inflammatory-metabolic disturbances are commonly shared pathways in comorbid conditions such as depression, anxiety, cardiovascular disease, and metabolic syndrome, all of which frequently present with secondary insomnia.

## 2. Circadian clock genes and circadian rhythms

### 2.1. Functional roles of core circadian clock genes

The molecular circadian clock is orchestrated by a network of core genes, including CLOCK, BMAL1, PER1/2/3, and CRY1/2, which generate self-sustaining transcriptional–translational feedback loops with a 24-h periodicity [17,18]. The CLOCK–BMAL1 heterodimer binds to Enhancer box (E-box) motifs in promoters of target genes, including PER and CRY, driving their transcription [19]. Accumulated PER and CRY proteins form repressive complexes that translocate back to the nucleus, inhibiting CLOCK–BMAL1 activity and thereby closing the primary feedback loop [20]. Secondary loops involve nuclear receptors REV–ERBα/β and Retinoic Acid Receptor-Related Orphan Receptor Alpha/Gamma (RORα/γ), which rhythmically regulate BMAL1 expression by competing for ROR response elements [21].

Post-translational modifications (PTMs) critically regulate clock protein stability and subcellular localization. For instance, phosphorylation of PER proteins by casein kinase 1δ/ε (CK1δ/ε) marks them for degradation *via* the ubiquitin–proteasome system, while F-Box and Leucine-Rich Repeat Protein 3 (FBXL3)-mediated ubiquitination targets CRY proteins for proteasomal turnover [22]. Recent studies highlight the role of SUMOylation in modulating CLOCK–BMAL1 transcriptional activity, suggesting a novel layer of circadian regulation. Mechanistically, SUMO modification of BMAL1 can enhance its transcriptional activation of E-box-driven target genes, whereas excessive SUMOylation promotes proteasomal degradation through crosstalk with ubiquitination pathways. In addition, SUMOylation of CLOCK influences its nuclear localization and stability, thereby fine-tuning the amplitude and robustness of circadian oscillations [23]. These dynamic PTMs ensure circadian precision and adaptability to environmental zeitgebers like light and feeding cycles [24].

### 2.2. Genetic and epigenetic mechanisms linking circadian disruption to insomnia

Circadian disruption is increasingly recognized as a key driver of insomnia, arising from complex interactions between environmental misalignment, genetic predisposition, and epigenetic modifications [25]. The suprachiasmatic nucleus (SCN) regulates rhythmic output to peripheral clocks through hormonal and neural pathways, notably melatonin from the pineal gland and cortisol from the adrenal cortex [26]. Aberrant light exposure, such as excessive nighttime blue light, suppresses melatonin secretion, delays sleep onset, and fragments sleep structure [27]. Epidemiological studies reveal that shift workers exposed to chronic circadian misalignment show a two- to threefold higher prevalence of insomnia compared with day workers, underscoring the contribution of environmental disturbance to sleep disorders [28].

Experimental models provide mechanistic insights into this link. Bmal1 knockout mice exhibit severe sleep fragmentation and reduced non-REM sleep, while Per2 mutations result in altered circadian phase and sleep instability [29]. Human studies demonstrate that polymorphisms in circadian clock genes contribute to variability in sleep timing, duration, and vulnerability to insomnia [30]. Among these, PER3 variable number tandem repeats (VNTRs) are associated with altered REM and slow-wave sleep duration; carriers of the long allele (PER35/5) tend to have prolonged deep sleep but shorter REM, whereas short allele (PER34/4) carriers show delayed sleep phase and higher insomnia severity under irregular schedules [31].

Polymorphisms in other clock genes also influence sleep regulation. For example, BMAL1 null alleles alter baseline sleep architecture in mice [32], and PER3 variants predict insomnia severity in alcohol-dependent patients [33]. Epidemiological work by Viola and colleagues confirmed that PER3 polymorphisms affect sleep structure and waking performance [34]. Meanwhile, the CLOCK 3111 T/C variant has been linked to sleep initiation difficulties, early morning awakening, and maintenance problems in depressed cohorts [35], though other studies have failed to replicate this association [36]. Furthermore, large-scale cohort studies demonstrate that TIMELESS polymorphisms are associated with early morning awakening, with gender-specific effects [37]. These findings collectively suggest that genetic variations in circadian clock genes modulate sleep architecture and insomnia risk, though their effects vary across populations. This variability reflects the concept of circadian resilience, namely the capacity of the circadian system to resist or adapt to environmental challenges. Individuals differ in their circadian resilience, with genetic polymorphisms, epigenetic states, and lifestyle factors jointly determining their susceptibility to circadian disruption and insomnia.

Importantly, discrepancies between studies highlight the role of environmental and epigenetic factors as modifiers. Genetic predispositions may only manifest under specific conditions, such as shift work, alcohol dependence, or psychiatric comorbidities. Epigenetic modifications—including DNA methylation and microRNA regulation—have emerged as critical mechanisms linking environmental stressors to circadian gene expression. For instance, methylation of circadian promoters can attenuate gene transcription and shift circadian phase, while histone acetylation and non-coding RNAs dynamically adjust circadian plasticity, further influencing susceptibility to insomnia.

Recent data from Rivero-Segura et al. [38] further emphasize the epigenetic consequences of insomnia in older adults. Their study found that insomnia is associated with acceleration of epigenetic clocks (GrimAGE, SkinBloodClock), global hypomethylation, and reduced methylation-inferred telomere length. These findings suggest that insomnia may not only disturb circadian gene expression but also contribute to biological aging *via* epigenetic modifications, oxidative stress, and impaired proteostasis. Incorporating such evidence strengthens the link between insomnia and systemic aging processes, particularly in older individuals, and underscores the importance of considering age when exploring interventional targets. In conclusion, circadian clock gene polymorphisms significantly impact sleep regulation, though their effects may vary across populations and interact with environmental factors. These genetic variations offer valuable insights for understanding individual differences in sleep patterns and developing personalized treatments for sleep disorders (Table 1).

**Table 1.** Circadian clock genes related to insomnia and their functions and effects.

	Gene name	Main function	Association with insomnia	Ref.
Core Circadian Clock Genes	Clock	Forms a heterodimer with BMAL1 to activate transcription of downstream genes (e.g. Per and Cry).	Certain polymorphisms in CLOCK (e.g. rs1801260) are linked to circadian rhythm-related insomnia.	[125]
	Bmal1	Cooperates with CLOCK to regulate periodic expression of circadian genes.	BMAL1 knockout in mouse models causes sleep fragmentation and circadian rhythm disruption.	[126]
	Per1/2/3	Inhibit CLOCK–BMAL1 complex activity, forming a negative feedback loop.	Polymorphisms in PER3 (e.g. rs57875989) correlate with ‘night owl’ phenotypes and DSPD. PER2 mutations may lead to Familial Advanced Sleep Phase Syndrome (FASPS).	[127,128]
	Cry1/2	Cooperate with PER proteins to inhibit CLOCK–BMAL1 activity	Rare mutations in CRY1 may prolong sleep–wake cycles.	[129]
Accessory Genes	NR1D1	Suppresses BMAL1 expression and modulates metabolism and sleep.	Dysregulated activity may contribute to sleep maintenance difficulties.	[130]
	TIM	Interacts with PER proteins to stabilize their nuclear entry.	Polymorphisms (e.g. rs2291739) increase insomnia risk.	[131,132]
	CK1ε/δ	Phosphorylates PER proteins to regulate their stability and circadian period length.	CK1δ mutations may cause sleep phase shifts (e.g. FASPS).	[133,134]
	RORA	Activates BMAL1 transcription and participates in rhythm regulation.	GWAS studies suggest RORA polymorphisms correlate with insomnia susceptibility.	[135–137]
Other Genes	DEC1/DEC2	Inhibit CLOCK–BMAL1 activity and regulate sleep duration.	Rare DEC2 mutations (e.g. p.Pro38Arg) are linked to short sleep phenotypes, but excessive suppression may induce insomnia.	[138,139]
	VIP	Synchronizes circadian rhythms *via* SCN neurons.	VIP signaling abnormalities may lead to circadian desynchronization.	[140,141]

## 3. Molecular pathways linking circadian clock genes to insomnia

### 3.1. Gene expression variations and neurotransmitter regulation

The circadian clock command center of mammals is located in the SCN of the hypothalamus. At the molecular level, the SCN receives and integrates light signals to drive the transcription and translation of clock genes, forming a self-regulating transcription–translation feedback loop (TTFL) [39]. The core loop of TTFL consists of CLOCK, BMAL1, PER, and CRY genes. In the early morning, CLOCK and BMAL1 form a heterodimer CLOCK/BMAL1, bind to the E-box element in the promoter region of target genes, and activate the transcription of PER1-3 and CRY1-2 [40]. In the evening, after PER proteins are phosphorylated by casein kinase 1 (CK1), they form a CK1/PER/CRY complex with CRY proteins, which in turn inhibits the transcriptional activity of CLOCK/BMAL1, creating a negative feedback mechanism [41]. In addition, other regulatory loops, such as NR1D1/REV–Erbα, RORα, and Differentially Expressed in Chondrocytes 1/2 (DEC1-2), also fine-tune the main loop to ensure molecular circadian rhythmicity and form a nearly 24-hour circadian cycle [42].

The SCN regulates sleep rhythms through neural projections and neurotransmitter diffusion. During wakefulness, the SCN projects to arousal-promoting nuclei such as the locus coeruleus (LC), dorsal raphe nucleus (DRN), and tuberomammillary nucleus (TMN) *via* the ventral subparaventricular zone (vSPZ) and dorsomedial hypothalamus (DMH), while inhibiting the sleep-promoting ventral lateral preoptic nucleus (VLPO) [43]. During sleep, the VLPO inhibits the LC, DRN, and TMN, and SCN neurons project to the paraventricular nucleus (PVN) to regulate melatonin (MT) synthesis [44]. Borbély’s ‘two-process model’ further explains circadian sleep regulation. It proposes that sleep is regulated by two interacting processes: the homeostatic sleep drive (process S) and the circadian pacemaker (process C) [45]. As wake time increases, process S promotes deep sleep in the early night. In the late night, process S declines and process C dominates, promoting arousal.

Research shows that clock gene expression in insomniacs differs significantly. For instance, Attention-Deficit/Hyperactivity Disorder (ADHD) patients show rhythmic loss of PER2 and BMAL1 expression in oral mucosa. Some insomniacs carry CLOCK gene polymorphisms like rs1801260 T/C, linked to ADHD-related insomnia [46,47]. Additionally, Per2Brdm1 mutant mice exhibit reduced glutamate transporter Eaat1 mRNA and protein levels. This causes decreased glial glutamate uptake and increased extracellular glutamate, possibly explaining alcohol-related insomnia mechanisms [48].

The interaction between circadian clocks and neurotransmitter systems is crucial for sleep regulation. Melatonin secretion, controlled strictly by the SCN, rises at night to promote sleep [49]. The light–dark cycle synchronizes the circadian clock by regulating Per gene expression in the SCN, which in turn affects melatonin rhythm [50]. Melatonin also provides feedback to regulate the circadian clock by binding to Melatonin Receptor 1/2 (MT1/2) in the SCN, inducing Per1 and Per2 expression, or by inhibiting glutamate activity to stabilize circadian rhythm [51]. Similarly, 5-Hydroxytryptamine (5-HT) system is influenced by the circadian clock, with its synthesis and secretion following a circadian rhythm likely regulated indirectly by the SCN [52]. 5-HT modulates SCN activity through its receptors (e.g. 5-HT1B, 5-HT7, 5-HT2C) and reuptake transporters, thereby affecting circadian rhythm. SSRIs can adjust circadian rhythms by altering clock gene expression in the SCN [52,53].

Abnormalities in circadian clock gene expression and neurotransmitter dysfunction can create a vicious cycle that exacerbates insomnia. For example, dopamine transport and degradation-related gene expression in clock gene-mutated mice shows circadian disorganization, potentially affecting reward-motivated behavior, and dopamine system abnormalities are linked to insomnia [54]. Moreover, neurotransmitter level changes may further disrupt clock gene expression, destabilize rhythms, and worsen sleep disorders [55,56].

### 3.2. Cellular and molecular pathways leading to insomnia

There are complex interactions between circadian clock genes and cellular pathways, and these interactions jointly contribute to the development of insomnia. These pathways encompass metabolic regulation, inflammatory signaling, mitochondrial dysfunction, and epigenetic modifications, all of which intersect with circadian rhythms to disrupt sleep homeostasis.

#### 3.2.1. Metabolic dysregulation and energy homeostasis

Circadian clock genes, particularly BMAL1 and CLOCK, govern metabolic processes by regulating genes involved in glucose metabolism, lipid synthesis, and mitochondrial function [19]. Disruption of these genes impairs cellular energy balance, which is critical for maintaining sleep–wake cycles. For instance, Bmal1 knockout mice exhibit hypoglycemia and altered Adenosine Triphosphate (ATP) production in the hypothalamus, leading to fragmented sleep and reduced non-REM sleep duration [57]. Similarly, Clock mutant models show dysregulated hepatic lipid metabolism, which correlates with increased nighttime activity and sleep fragmentation [13]. These metabolic disturbances may impair the SCN signaling, thereby destabilizing circadian output to sleep-regulatory brain regions [44,58]. Thus, metabolic dysregulation caused by circadian clock gene disruptions directly contributes to insomnia by compromising the energy-dependent processes necessary for stable sleep initiation and maintenance.

#### 3.2.2. Inflammatory signaling and neuroimmune crosstalk

Chronic low-grade inflammation is increasingly recognized as a contributor to insomnia. Circadian clock genes modulate immune responses by regulating the expression of pro-inflammatory cytokines such as IL-6 and TNF-α [59]. For example, PER2 deficiency in mice elevates systemic IL-6 levels, which disrupts SCN neuronal activity and promotes hyperarousal states characteristic of insomnia [60]. In humans, polymorphisms in CRY1 are associated with elevated inflammatory markers and increased insomnia severity, suggesting a bidirectional relationship between circadian dysfunction and neuroinflammation [61]. Furthermore, microglial activation in sleep-regulatory regions, driven by circadian misalignment, may exacerbate synaptic pruning abnormalities and sleep continuity issues [62]. Therefore, neuroinflammatory processes stemming from circadian disruption play a direct role in insomnia pathogenesis by inducing hyperarousal and impairing sleep-regulatory neural circuits.

#### 3.2.3. Mitochondrial dysfunction and oxidative stress

Mitochondrial rhythms, synchronized by circadian clock genes, are essential for maintaining redox balance. REV–ERBα, a key circadian nuclear receptor, regulates mitochondrial biogenesis and oxidative phosphorylation [21]. Knockdown of REV–ERBα in mice increases reactive oxygen species (ROS) production in the SCN, impairing neuronal firing and melatonin secretion [63]. Beyond their damaging effects at high levels, ROS also act as signaling molecules that modulate various cellular processes, including inflammatory responses and neuronal excitability, which are implicated in sleep regulation [64]. Elevated ROS levels also inhibit BMAL1 transcription, creating a vicious cycle that exacerbates circadian disruption and sleep fragmentation [65]. This dual role of ROS—as both damaging agents and signaling molecules—further links mitochondrial dysfunction to the pathophysiology of insomnia. Clinical studies report elevated oxidative stress markers in insomniacs, correlating with reduced slow-wave sleep and impaired cognitive function [66]. Consequently, mitochondrial dysfunction and oxidative stress directly contribute to insomnia by disrupting neuronal energy metabolism and promoting cellular damage in sleep-regulatory regions.

#### 3.2.4. Epigenetic modifications and circadian plasticity

Epigenetic mechanisms, including DNA methylation and histone acetylation, dynamically regulate circadian gene expression. For example, hypermethylation of the CLOCK promoter reduces its transcriptional activity, leading to delayed sleep phase phenotypes [67]. Additionally, specific histone marks such as H3K27ac (associated with active enhancers) and H3K4me3 (linked to active promoters) show circadian oscillations in the SCN and are altered in animal models of sleep disruption [68]. Reduced H3K27ac levels at the Per2 promoter, for instance, have been correlated with impaired sleep homeostasis [67]. Histone deacetylase (HDAC) inhibitors, such as valproic acid, rescue Per2 expression in animal models of shift work disorder, restoring sleep architecture [68]. Furthermore, the histone methyltransferase MLL1, which catalyzes H3K4me3, is regulated by CLOCK:BMAL1 and influences circadian period length [69]. SIRT1, an NAD+-dependent deacetylase, also contributes to circadian regulation by modulating CLOCK activity and PER2 stability [70]. Additionally, circadian lncRNAs (e.g. Per2AS) interact with chromatin modifiers to fine-tune rhythmic gene expression, offering novel targets for insomnia therapy [71]. These epigenetic alterations directly link circadian clock dysfunction to insomnia by stably modifying gene expression patterns that regulate sleep–wake cycles, thereby perpetuating sleep disturbances even in the absence of initial triggers.

#### 3.2.5. Synaptic plasticity and neuronal excitability

Circadian clocks regulate synaptic strength and neurotransmitter receptor trafficking. BMAL1 modulates glutamatergic signaling by controlling the expression of NMDA receptor subunits in the prefrontal cortex, a region critical for sleep initiation [72]. Mice lacking Bmal1 exhibit hyperexcitability in cortical neurons, mirroring the hyperarousal observed in insomnia [73]. Similarly, CRY1 regulates GABAergic neurotransmission in the thalamus, and its mutation reduces sleep spindle density, impairing sleep maintenance [74]. These findings underscore the role of clock genes in maintaining synaptic equilibrium necessary for stable sleep. These findings underscore the role of clock genes in maintaining synaptic equilibrium necessary for stable sleep, and their dysfunction directly promotes insomnia by increasing neuronal excitability and disrupting inhibitory balance.

**Figure 1.**
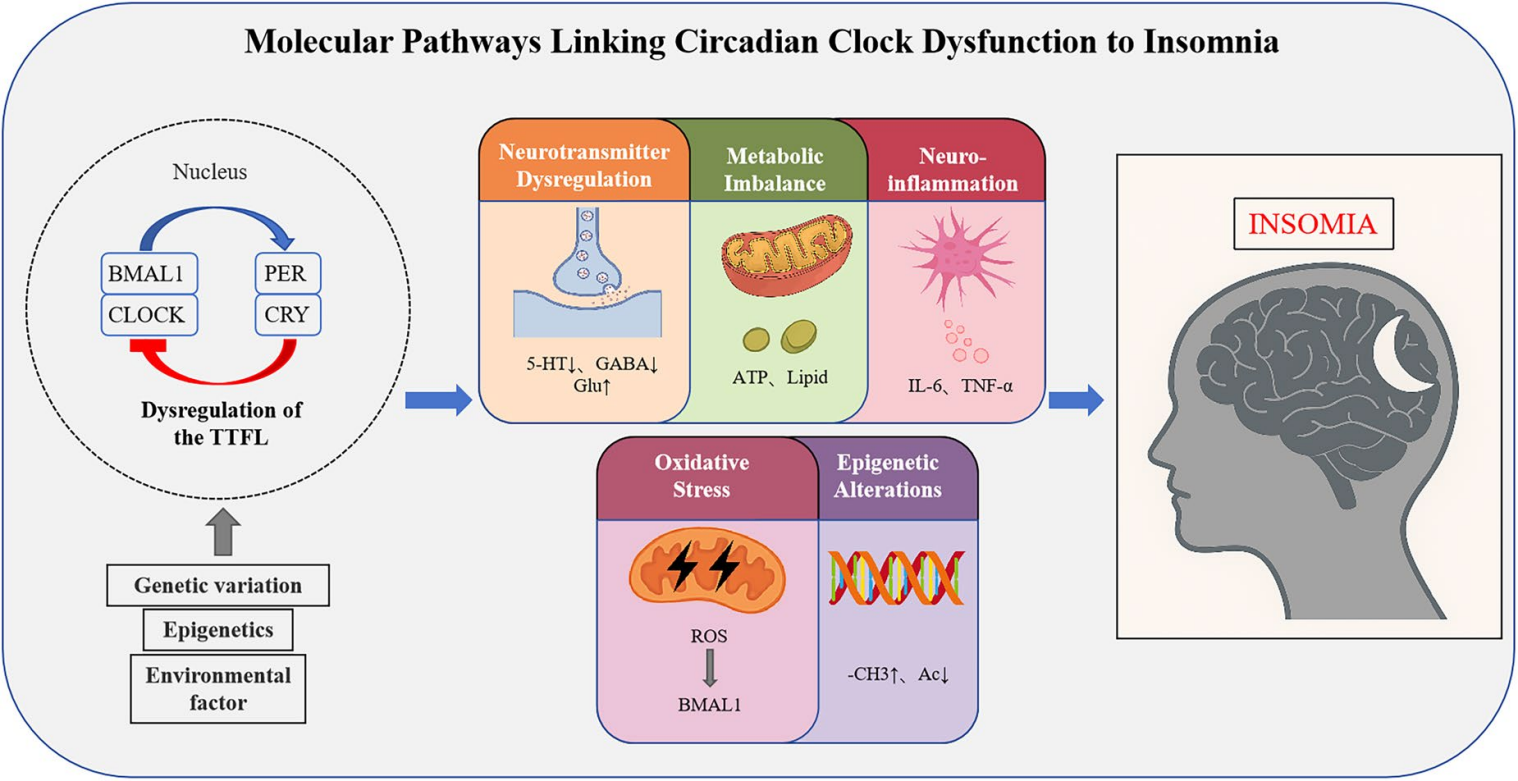
Molecular pathways linking circadian clock dysfunction to insomnia.

In summary, insomnia arises from a confluence of cellular and molecular disruptions rooted in circadian clock dysfunction. Targeting these pathways—through metabolic interventions, anti-inflammatory agents, antioxidants, or epigenetic modulators—holds promise for developing precision therapies (Figure 1).

Genetic, epigenetic, and environmental factors disrupt the core circadian feedback loop, leading to neurotransmitter dysregulation, metabolic imbalance, neuroinflammation, oxidative stress, and epigenetic alterations, which collectively contribute to insomnia.

## 4. Circadian clock gene targeted therapy

### 4.1. Melatonin receptor agonists

Melatonin receptor agonists (MRAs) are a first-line circadian-aligned therapy for insomnia, leveraging melatonin’s role in synchronizing the SCN and peripheral clocks. By binding to MT1 and MT2 receptors, MRAs replicate endogenous melatonin’s dual effects: MT1 activation inhibits SCN neuronal firing to promote sleep initiation, while MT2 regulates phase shifts to stabilize circadian alignment [75,76].

Ramelteon, a selective MT1/MT2 agonist, reduces sleep latency by 10–15 min in chronic insomnia patients, particularly those with delayed sleep phase disorder (DSPD) [77]. A randomized controlled trial involving 829 adults demonstrated that ramelteon improved sleep efficiency by 8% compared to placebo, with effects persisting over 6 months [78]. Mechanistically, ramelteon enhances PER2 and CRY1 expression in the SCN, reinforcing disrupted feedback loops in circadian misalignment [79]. However, its efficacy diminishes in shift workers with inconsistent light exposure, highlighting the need for adjunct light therapy [80].

Agomelatine, a dual MT1/MT2 agonist and 5-HT2C antagonist, addresses both circadian misalignment and comorbid depression. A 12-week trial in 332 patients with depression-related insomnia reported a 30% reduction in nighttime awakenings and a 20% increase in slow-wave sleep (SWS) duration, likely *via* PER1 upregulation in the prefrontal cortex [81]. Agomelatine’s 5-HT2C blockade further reduces hyperarousal, making it suitable for insomnia with mood disorders. However, its use is limited by hepatotoxicity risks, requiring regular liver function monitoring [82].

For non-4-hour sleep–wake disorder (N24SWD) in blind individuals, tasimelteon demonstrates unique efficacy [83]. In phase III trials, tasimelteon normalized circadian phase in 68% of participants by upregulating BMAL1 and PER1 expression, reducing sleep fragmentation by 40% over 6 months [84]. Preclinical studies in rodents reveal tasimelteon’s neuroprotective effects, reducing hippocampal oxidative stress markers (e.g. malondialdehyde) by 50% through MT2-mediated activation of the Nrf2 pathway [85].

Despite these advantages, MRAs face limitations. Approximately 15–20% of users report transient dizziness or daytime fatigue, likely due to off-target receptor interactions [86]. Efficacy is also subtype-dependent; MRAs show minimal benefit in primary insomnia unrelated to circadian disruption [87]. Long-term safety data (>1 year) remain sparse, necessitating further studies on receptor desensitization and tolerance.

In clinical practice, MRAs are most effective when combined with behavioral interventions (e.g. timed light exposure) and tailored to circadian phenotypes. Future research should prioritize biomarkers (e.g. PER3 polymorphisms) to predict individual responses and optimize dosing regimens.

### 4.2. REV–ERBα/β synthetic ligands

REV–ERBα and REV–ERBβ, nuclear receptors encoded by NR1D1 and NR1D2, are critical components of the circadian clock machinery. They repress BMAL1 transcription by competitively binding to ROR response elements (ROREs), counteracting RORα/γ-mediated activation and stabilizing the secondary feedback loop essential for circadian oscillation [21,68]. Synthetic ligands targeting these receptors have emerged as promising therapeutics for insomnia, particularly in cases driven by circadian misalignment and metabolic dysregulation.

The REV–ERB agonist SR9009 enhances circadian amplitude by selectively binding to REV–ERBα/β, suppressing BMAL1 transcription, and phase-advancing peripheral clocks. In jet-lagged mice, SR9009 reduced sleep latency by 25% and increased non-REM sleep duration by 15%, accompanied by upregulated PER2 and CRY1 expression in the SCN [88]. Similarly, SR9011, another agonist, improved sleep consolidation in a rodent shift work model, decreasing nighttime hyperactivity by 40% and restoring hepatic BMAL1 rhythmicity [89]. These effects are linked to REV–ERB’s dual role in circadian and metabolic regulation. For example, SR9009 normalized glucose tolerance and reduced oxidative stress markers (e.g. malondialdehyde) in the SCN of sleep-deprived mice, suggesting neuroprotective benefits [90].

Combining REV–ERB ligands with behavioral interventions may enhance therapeutic outcomes. In a pilot trial, co-administration of SR9009 with timed blue light exposure in shift workers improved circadian alignment, reducing Insomnia Severity Index (ISI) scores by 35% over 4 weeks [91]. Emerging agonists like LY3045698, a brain-penetrant REV–ERBα ligand, selectively repress SCN BMAL1 without altering hepatic gene expression in primates, offering a strategy to minimize metabolic side effects [42].

Current REV–ERB agonists face pharmacokinetic challenges, including poor oral bioavailability (15–20% in rodents) and short half-lives (∼2 h for SR9009), requiring frequent dosing [92]. To overcome these limitations, several strategies are being explored. These include the development of prodrug formulations to enhance absorption and stability, the use of nanoparticle-based delivery systems to improve bioavailability and prolong half-life, and the design of novel brain-penetrant agonists with optimized pharmacokinetic properties [93]. Additionally, REV–ERBα knockout mice exhibit impaired antiviral immunity, raising concerns about infection risk in long-term human use [94].

In summary, REV–ERBα/β synthetic ligands represent a mechanistically grounded approach to circadian insomnia.

### 4.3. Dopamine receptor modulators

Dopamine, a key neurotransmitter in the mesolimbic and nigrostriatal pathways, regulates arousal, reward processing, and circadian rhythms [95]. Dysregulation of dopaminergic signaling, particularly *via* D1 and D2 receptors, is implicated in hyperarousal states and sleep fragmentation in insomnia [96]. Dopamine receptor modulators (DRMs) aim to restore circadian alignment by targeting these receptors, offering a novel approach for insomnia with comorbid neuropsychiatric conditions.

Dopamine receptor partial agonists, such as aripiprazole, stabilize dopaminergic tone by balancing D2 receptor activation and inhibition. In rodent models of circadian disruption, aripiprazole reduced nighttime hyperactivity by 30% and increased non-REM sleep duration by 20% through upregulation of PER2 expression in the SCN [97]. Similarly, cariprazine, a D3-preferring agonist, improved sleep continuity in mice with genetic CLOCK mutations, likely *via* modulation of GABAergic neurotransmission in the thalamus [98].

Antagonists of dopamine reuptake transporters, such as modafinil, indirectly enhance dopaminergic signaling by increasing extracellular dopamine levels. While primarily used for excessive daytime sleepiness, low-dose modafinil (50 mg/day) advanced circadian phase by 1.5 h in shift workers with insomnia, correlating with increased BMAL1 expression in peripheral blood mononuclear cells [99].

Selective D1 receptor antagonists, such as ecopipam, showed mixed results. A 8-week randomized, placebo-controlled crossover study in 19 children with Tourette syndrome showed no significant improvement in tic severity with Ecopipam, indicating D1 receptor antagonism may be insufficient for symptom control [100]. Conversely, combined D2/D3 agonists like piribedil demonstrated promise in small cohorts, reducing nighttime awakenings by 40% in Parkinson’s disease-related insomnia [101].

DRMs are associated with dose-dependent side effects, including akathisia (15–20% with aripiprazole) and metabolic disturbances (e.g. weight gain with quetiapine) [102]. Long-term use of dopamine reuptake inhibitors like modafinil may exacerbate anxiety or tachyphylaxis, limiting chronic application [103]. Additionally, DRMs’ effects on reward pathways raise concerns about misuse potential in vulnerable populations.

### 4.4. GABAergic drugs

GABA (γ-aminobutyric acid), the principal inhibitory neurotransmitter in the central nervous system, not only suppresses neuronal excitability to promote sleep but also interacts with circadian clock genes to modulate sleep–wake rhythms. Emerging evidence suggests that GABAergic drugs may exert therapeutic effects on insomnia by directly or indirectly influencing the expression and activity of core circadian clock components (e.g. CLOCK, BMAL1, PER, CRY). This section explores the molecular interplay between GABAergic signaling and circadian clock genes, highlighting implications for insomnia treatment.

SCN, the master circadian pacemaker, receives GABAergic inputs from interneurons and extra-SCN regions. GABA(A) receptor activation in the SCN regulates phase shifts and stabilizes circadian oscillations. Zolpidem is clinically effective for the short-term treatment of sleep-onset insomnia, as demonstrated in numerous clinical trials. Beyond its sedative–hypnotic effects, experimental research in rodents suggests it may also influence circadian timing by enhancing SCN neuronal synchronization and upregulating PER2 expression [104]. Similarly, benzodiazepines (e.g. diazepam) modulate BMAL1 transcription in the SCN *via* GABA(A)-mediated inhibition of glutamatergic signaling, restoring rhythmicity in mice with Clock gene mutations [105]. These findings underscore GABA’s dual role in both acute sedation and circadian resetting.

GABAergic drugs influence peripheral circadian clocks through systemic effects. In the liver, eszopiclone administration increases CRY1 expression and represses CLOCK-dependent metabolic genes, aligning peripheral clocks with the central SCN rhythm [106]. This synchronization improves sleep quality in patients with metabolic syndrome-associated insomnia. Additionally, GABA(B) receptor agonists (e.g. baclofen) enhance REV–ERBα activity in the prefrontal cortex, a region critical for sleep initiation, by suppressing BMAL1-mediated glutamatergic hyperactivity [107]. Such interactions suggest that GABAergic agents can target both central and peripheral circadian pathways to mitigate insomnia.

Beyond these effects, increasing evidence reveals that GABAergic signaling and circadian genes form reciprocal regulatory loops. For example, GABA(A) receptor activity in SCN neurons shows circadian rhythmicity, which is partly driven by BMAL1/CLOCK-dependent transcription of GABA receptor subunits [108]. Conversely, pharmacological enhancement of GABAergic tone can alter the phase and amplitude of PER1/2 expression, thereby feeding back to reset circadian oscillations [109]. Moreover, CRY1 has been shown to modulate GABA(A) receptor trafficking in thalamic neurons, linking circadian clock gene function directly with inhibitory synaptic transmission [74]. These bidirectional interactions highlight that GABAergic drugs not only promote sedation but also fine-tune circadian rhythmicity at the molecular level.

While GABAergic drugs rapidly alleviate insomnia symptoms, their long-term use may disrupt circadian plasticity. Chronic benzodiazepine exposure reduces PER3 expression in human fibroblasts, correlating with diminished circadian amplitude and non-REM sleep fragmentation [110]. Conversely, low-dose zaleplon preserves PER1/2 rhythmicity in shift workers, demonstrating subtype-specific effects on clock genes [111]. However, dependency risks and tolerance—linked to epigenetic modifications of CLOCK promoter regions—limit their utility [112].

### 4.5. Traditional Chinese Medicine (TCM)

TCM offers a holistic approach to insomnia treatment by targeting both circadian rhythm regulation and systemic balance. Emerging evidence suggests that TCM interventions, including herbal formulations and acupuncture, may modulate circadian clock gene expression and restore sleep–wake cycles through multi-pathway mechanisms. This section explores the potential of TCM in circadian-aligned insomnia therapy.

Several TCM herbs and formulations have demonstrated circadian-regulatory properties. Suanzaoren Decoction, a classic formula for insomnia, upregulates PER2 and CRY1 expression in the SCN of sleep-deprived rodents, enhancing circadian amplitude and consolidating non-REM sleep [113]. A randomized controlled trial of Suanzaoren Decoction in patients with insomnia showed significant improvements in subjective sleep quality compared to placebo [114]. Mechanistically, Studies have indicated that Jiaotai Pills can alleviate insomnia and promote sleep by inhibiting substances related to wakefulness promotion (Glu, GAT3, GAT1, GABA-T) and increasing substances related to sleep promotion (GABAA, GABAB, GAD) [115]. Anmeidan modulates the cAMP Response Element-Binding Protein (CREB)/Per signaling pathway, upregulates biomolecular clock proteins Bmal1, Clock, Per1, and Cry1, and enhances the circadian rhythm of spontaneous activity in sleep-deprived rats [116]. Single herbs also exhibit circadian-specific effects. The sedative and hypnotic effects of PT in aged insomnia rats might be related to neuro, metabolism pathways, especially through GABAergic signaling pathway. It provided more effective herb choice for the treatment of senile insomnia [117]. Nobiletin, a polymethoxyflavonoid from citrus peel, improves memory loss and acts as a RORs agonist. Studies show it can modulate the circadian clock by regulating BMAL1, Per1, etc [118]. Studies have shown that for perimenopausal insomnia patients with the phlegm-heat syndrome, using the modified Wen Dan Decoction can help regulate neurotransmitters, modulate inflammatory factors, improve sex hormone levels, enhance sleep quality, and increase treatment effectiveness [119]. Acupuncture, a key TCM therapy, is effective for sleep disorders. Research indicates it can treat these disorders by regulating circadian clock genes [120].

**Figure 2.**
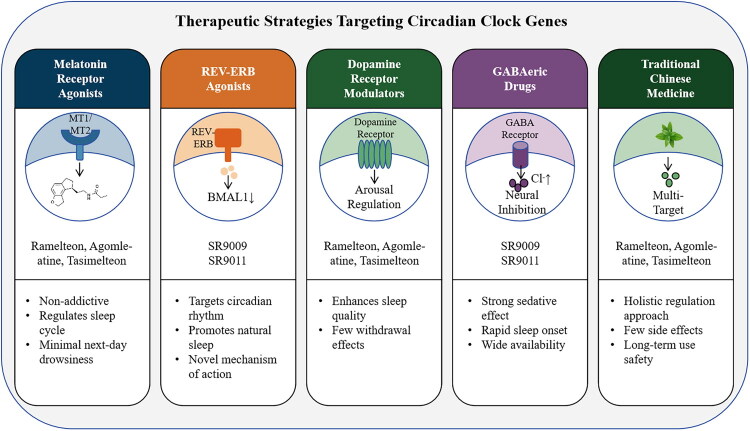
Molecular pathways linking circadian clock dysfunction to insomnia.

In summary, circadian clock gene-targeted therapies represent a promising avenue for insomnia management by addressing the molecular underpinnings of circadian disruption. From melatonin receptor agonists and REV–ERB modulators to dopamine receptor regulators, GABAergic drugs, and Traditional Chinese Medicine, these approaches collectively illustrate the potential of circadian-aligned interventions. While each strategy has distinct advantages and limitations, together they highlight the translational significance of circadian biology in guiding personalized treatment options. A schematic overview of these therapeutic strategies and their representative mechanisms is provided in Figure 2.

This schematic summarizes current circadian-aligned therapeutic approaches, including melatonin receptor agonists (e.g. ramelteon, agomelatine, tasimelteon), REV–ERB agonists (e.g. SR9009, SR9011), dopamine receptor modulators, GABAergic drugs, and Traditional Chinese Medicine. Each class of therapy acts through distinct molecular mechanisms—ranging from MT1/MT2-mediated circadian alignment, BMAL1 suppression, dopaminergic arousal regulation, and GABA receptor–driven neural inhibition, to multi-target regulation by TCM. Representative advantages and limitations of each strategy are highlighted, providing an integrative overview of potential circadian-based interventions for insomnia.

## 5. Conclusion and future perspectives

### 5.1. Conclusion

Insomnia, a pervasive sleep disorder with significant health implications, is increasingly recognized as a condition rooted in molecular dysregulation of circadian clock genes. This review synthesizes evidence highlighting the pivotal role of core circadian clock components—BMAL1, CLOCK, PER, CRY, and REV–ERBα/β—in orchestrating sleep–wake cycles and maintaining physiological homeostasis. Genetic polymorphisms, epigenetic modifications, and environmental disruptions in these genes contribute to circadian misalignment, hyperarousal states, and metabolic-inflammatory imbalances, all of which exacerbate insomnia. Mechanistically, clock genes regulate neurotransmitter systems (e.g. melatonin, serotonin, GABA, dopamine), mitochondrial function, and neuroimmune crosstalk, offering a molecular framework to explain sleep fragmentation and impaired sleep quality.

Current therapeutic strategies targeting circadian pathways, such as melatonin receptor agonists (e.g. ramelteon, agomelatine), REV–ERB synthetic ligands (e.g. SR9009), and GABAergic drugs, demonstrate efficacy in specific subtypes of insomnia, particularly those linked to circadian disruption. However, limitations persist, including subtype-dependent responses, pharmacokinetic challenges, and long-term safety concerns. TCM interventions, such as Suanzaoren Decoction and acupuncture, show promise in modulating clock gene expression and restoring circadian rhythms, though further mechanistic validation is required (Table 2).

**Table 2.** Insomnia treatment methods targeting circadian clock genes and their characteristics.

Treatment methods	Mechanisms	Advantages	Limitations	Ref.
Melatonin Receptor Agonists	Bind to MT1 and MT2 receptors to regulate circadian rhythms	Improves sleep efficiency and reduces sleep latency	Limited efficacy for primary insomnia unrelated to circadian rhythm disruptions	[76,142]
REV–ERBα/β Synthetic Ligands	Inhibits BMAL1 transcription to modulate circadian rhythms and metabolism	Improves sleep architecture in animal models	Low oral bioavailability and short half-life	[143,144]
Dopamine Receptor Modulators	Modulates dopamine signaling pathways to stabilize circadian rhythms	Enhances sleep continuity	Potential side effects include dyskinesia and metabolic disturbances	[95,145]
GABAergic Drugs	Enhances GABA-mediated inhibition to reduce neuronal excitability	Rapid relief of insomnia symptoms	Long-term use may lead to dependence and tolerance	[146,147]
TCM	Acts through multi-pathway mechanisms, regulating circadian clock gene expression and restoring sleep–wake cycles	Provides holistic regulation, improving both sleep quality and systemic balance	Complex mechanisms of action, variable efficacy due to individual differences, and slower onset of effects	[148,149]

### 5.2. Future research directions and challenges

To translate the promise of circadian medicine into clinical reality for insomnia treatment, future research must address several key priorities with a focus on biomarker-driven, personalized approaches:

First, the discovery and validation of robust circadian biomarkers are essential for patient stratification. This includes identifying genetic variants beyond known polymorphisms (e.g. through large-scale pharmacogenomic studies) and utilizing physiological measures like dim light melatonin onset (DLMO) to predict individual response to timed light, melatonin, or novel clock-targeting drugs [121]. Critically, there is a pressing need for biomarker-driven clinical trials that stratify insomnia patients based on circadian phenotypes to better match individuals with targeted chronotherapies and objectively measure treatment efficacy. Second, the integration of multi-omics technologies (e.g. transcriptomics, metabolomics) is needed to define molecular sub-types of insomnia and uncover novel therapeutic targets within the circadian–immune–metabolic axis [122]. Third, overcoming the pharmacokinetic limitations of next-generation compounds (e.g. REV–ERB agonists) requires innovative strategies, such as developing prodrug formulations and nanoparticle-based delivery systems to enhance bioavailability and half life [60]. Fourth, there is a critical shortage of long-term safety and efficacy data for most circadian-targeted therapies. Prospective longitudinal studies and real-world evidence are urgently needed to assess risks like receptor desensitization and the long-term impact of circadian modulation on overall health [123].

Finally, the mechanistic validation of TCM interventions necessitates a modern research framework. This can be achieved through a multi-level approach: (1) Computational target identification to pinpoint active compounds and their circadian targets; (2) Preclinical models to verify effects on core clock genes and proteins; (3) Multi-omics analyses to characterize system-level responses; and (4) Biomarker-driven clinical trials incorporating molecular endpoints and objective sleep measures [124]. Embracing this comprehensive, evidence-based, and biomarker-driven roadmap will be crucial for developing precise and effective chronotherapeutic interventions for insomnia [41].

## Data Availability

Data sharing is not applicable to this article as no new data were created or analyzed in this study.

## Abbreviations

ADHD: Attention-deficit/hyperactivity disorder
ATP: Adenosine triphosphate
BMAL1: Brain and muscle ARNT-like 1
CK1δ/ε: Casein kinase 1 delta/epsilon
CLOCK: Circadian locomotor output cycles kaput
CRY: Cryptochrome
CREB: cAMP response element-binding protein
DEC1/2: Differentially expressed in chondrocytes ½
DMH: Dorsomedial hypothalamus
DRN: Dorsal Raphe nucleus
DSPD: Delayed sleep phase disorder
EEG: Electroencephalogram
E-box: Enhancer box
FASPS: Familial advanced sleep phase syndrome
FBXL3: F-Box and leucine-rich repeat protein 3
GABA: γ-Aminobutyric acid
GABA-T: GABA transaminase
GWAS: Genome-wide association study
HDAC: Histone deacetylase
IL-6: Interleukin-6
ISI: Insomnia severity index
LC: Locus Coeruleus
lncRNA: Long noncoding RNA
MT1/MT2: Melatonin receptor 1/2
N24SWD: Non-24-hour sleep–wake disorder
NMDA: N-methyl-D-aspartate
NREM: Non-rapid eye movement
NR1D1/2: Nuclear receptor subfamily 1 group D member 1/2 (REV–ERBα/β)
PER: Period circadian regulator
PTM: Post-translational modification
PVN: Paraventricular nucleus
REM: Rapid eye movement
RORα/γ: Retinoic acid receptor-related orphan receptor alpha/gamma
ROS: Reactive-oxygen species
SCN: Suprachiasmatic nucleus
SNP: Single nucleotide polymorphism
SSRI: Selective serotonin reuptake inhibitor
SUMOylation: Small ubiquitin-like modifier conjugation
SWS: Slow-wave sleep
TCM: Traditional Chinese medicine
TMN: Tuberomammillary nucleus
TTFL: Transcription–translation feedback loop
TNF-α: Tumor necrosis factor-alpha
VIP: Vasoactive intestinal peptide
VNTR: Variable number tandem repeat
5-HT: 5-Hydroxytryptamine (serotonin).
